# When all children comprehend: increasing the external validity of narrative comprehension development research

**DOI:** 10.3389/fpsyg.2014.00168

**Published:** 2014-03-13

**Authors:** Silas E. Burris, Danielle D. Brown

**Affiliations:** Department of Psychology, Howard UniversityWashington, DC, USA

**Keywords:** narrative comprehension, development, knowledge integration, goal structure understanding, causal inferences, external validity, review

## Abstract

Narratives, also called stories, can be found in conversations, children's play interactions, reading material, and television programs. From infancy to adulthood, narrative comprehension processes interpret events and inform our understanding of physical and social environments. These processes have been extensively studied to ascertain the multifaceted nature of narrative comprehension. From this research we know that three overlapping processes (i.e., knowledge integration, goal structure understanding, and causal inference generation) proposed by the constructionist paradigm are necessary for narrative comprehension, narrative comprehension has a predictive relationship with children's later reading performance, and comprehension processes are generalizable to other contexts. Much of the previous research has emphasized internal and predictive validity; thus, limiting the generalizability of previous findings. We are concerned these limitations may be excluding underrepresented populations from benefits and implications identified by early comprehension processes research. This review identifies gaps in extant literature regarding external validity and argues for increased emphasis on externally valid research. We highlight limited research on narrative comprehension processes in children from low-income and minority populations, and argue for changes in comprehension assessments. Specifically, we argue both on- and off-line assessments should be used across various narrative types (e.g., picture books, televised narratives) with traditionally underserved and underrepresented populations. We propose increasing the generalizability of narrative comprehension processes research can inform persistent reading achievement gaps, and have practical implications for how children learn from narratives.

The human mind constantly interprets and learns new information from narratives, commonly referred to as stories. The majority of our conversations, media, and early educational resources occur as narrative discourse (Graesser et al., 1991, 1997, 2002b; Tannock et al., 1993; Trabasso, 1994; Berger, 1997; Trabasso and Stein, 1997; Pearce, 2003; Cevasco and van den Broek, 2008). Narratives are typically stories that contain characters, their goals, and successive events leading to accomplishment of goals (Trabasso, 1994; Eaton et al., 1999; Magliano and Radvansky, 2001). Narratives are often experienced as aural presentations (e.g., verbal stories), wordless picture sequences (e.g., young children's picture books), audio-visual films (e.g., movies), and written text (Trabasso et al., 1992; Berman and Slobin, 1994; Graesser et al., 2003; Paris and Paris, 2003; Berman, 2004; Kendeou et al., 2005, 2008; Verhallen et al., 2006). Research suggests that all narrative forms require similar underlying processes for comprehension (Trabasso and Nickels, 1992; Eaton et al., 1999; Graesser et al., 2002a; Paris and Paris, 2003; Kendeou et al., 2005, 2008; Florit et al., 2011).

Precursors to narrative comprehension processes emerge during infancy (Bauer and Shore, 1987; Bauer, 1992; Wenner and Bauer, 1999; Henderson and Woodward, 2011; Gerson and Woodward, 2012) and improve with narrative exposure, familiarity, and adult support (Morrow, 1985; Bauer and Shore, 1987; Berman, 1995; Whitehurst and Lonigan, 1998). These processes help children interpret external information to understand physical and social environments (Bauer, 1992; Berger, 1997) and encourage later school-related skills (Brown et al., 1986, 2011; Bauer et al., 1999; Paris and Paris, 2003; Pelletier and Astington, 2004; Kendeou et al., 2005, 2008; van den Broek et al., 2005; Zucker et al., 2010). Studies have even suggested a connection between narrative exposure and vocabulary development (Trostle and Hicks, 1998; Whitehurst and Lonigan, 1998; Brown et al., 2011; see Lynch et al., 2008 for a different perspective). Potential benefits of narrative exposure have inspired many researchers to explore the multifaceted nature of narrative comprehension. These explorations, however, have been limited by their emphasis on internal and predictive validity rather than external validity and generalizability.

This review addresses the external validity of research on narrative comprehension development as described by the constructionist paradigm (Graesser et al., 1994). First, we describe the constructionist paradigm's perspective on narrative comprehension, which argues that comprehension processes are employed to construct meaningful mental representations of narratives. Second, we review literature on the development of narrative comprehension processes from infancy into adulthood. We identify gaps in this research for specific populations and ages. Third, we compare and discuss on-line and off-line assessments of narrative comprehension processes and propose a multi-method approach for developmental studies. Last, we argue that future research must incorporate methodology and scope that intentionally assesses narrative comprehension across diverse populations, knowledgebases, and media. Empirically informing our understanding of the generalizability of comprehension processes development will guide researchers to more accurately assess children and narrative media in the future.

## Narrative comprehension: the constructionist paradigm

The constructionist paradigm defines narrative comprehension as a coherent understanding resulting from overlapping processes to form a comprehender's mental representation (Graesser et al., 1994, 1997, 2002a; van den Broek et al., 1996, 2005; Graesser and Wiemer-Hastings, 1999). Asserting that comprehenders “search after meaning” within narratives, Graesser and colleagues identified integration of prior knowledge (Graesser et al., 1997, 2003; Best et al., 2008), goal structure understanding (Graesser et al., 1994), and generation of causal inferences (Trabasso and Nickels, 1992) as overlapping processes that assist the maintenance of relevant story details in memory (Trabasso et al., 1989; Bower and Morrow, 1990; Zwaan et al., 1995a; Zwaan and Radvansky, 1998; Kurby and Zacks, 2012). Comprehension successfully occurs when these processes converge as a meaningful and coherent mental representation (Kintsch, 1988; van den Broek et al., 2005).

Other theories include processes described by the constructionist paradigm (e.g., inference generation, the integration of prior knowledge); however, they typically emphasize a more narrowed approach and would benefit from an increased focus on external validity and generalizability (e.g., Kintsch, 1988; McKoon and Ratcliff, 1992). The holistic view proposed by the constructionist paradigm emphasizes relations between processes and can be developmentally examined. Although developmental research on narrative comprehension and narrative production are clearly different, these differences are not always made explicit in the literature. For example, storytelling procedures can assess both comprehension and production abilities. It is the method of analyzing these storytelling procedures which clarifies the distinction. Studies of production are often interested in children's or adults' narrative quality, length, details, and cohesion (Berman, 1988, 2004; Shapiro and Hudson, 1991; Berman and Slobin, 1994; Peterson and McCabe, 1994; Wigglesworth, 1997; Peterson et al., 1999; Kulkofsky et al., 2008; McCabe et al., 2008; Curenton, 2010), whereas studies of comprehension examine mechanisms underlying the construction of narrative mental representations (Paris and Paris, 2003). This review identifies trends in the comprehension research field and describes them in Table 1 (i.e., processes investigated according to population demographics). These trends reveal external validity concerns of population exclusion in comprehension development research. We discuss each process's role in comprehension, beginning with the integration of prior knowledge.

**Table 1:** **Numbers of sources describing narrative comprehension processes across populations from low- and middle- to high-income households**.

**SES × age**	**0–1 years**	**1–2 years**	**2–3 years**	**3–4 years**	**4–5 years**	**5–9 years**	**9–17 years**
**KNOWLEDGE INTEGRATION**							
Middle- to high-income households	0	0	0	0	1	6	4
Low-income households	0	0	0	0	0	0	0
**GOAL STRUCTURE UNDERSTANDING**							
Middle- to high-income households	3	5	0	7	19	24	16
Low-income households	0	0	0	2	8	5	2
**CAUSAL INFERENCE GENERATION**							
Middle- to high-income households	3	5	1	3	13	19	11
Low-income households	0	0	1	1	5	2	2

This table uses 71 sources with child samples from this review that describe narrative comprehension processes across childhood. Sources observing multiple processes or children from a range of socioeconomic statuses were listed more than once to account for all populations represented. For a more detailed list of references, see Appendices A and B.

### Knowledgebase integration

The process of knowledge integration requires access to stores of generic world knowledge and personal experiences in order to build narrative mental representations (Gowie, 1973; Kintsch, 1988; Graesser et al., 1994, 1997, 2001, 2002a,b; Graesser and Wiemer-Hastings, 1999; Brandão and Oakhill, 2005; Gerrig, 2011). Prior knowledge compensates for gaps in narrative coherence or when information is ambiguous (McNamara and Kintsch, 1996; Graesser et al., 2001, 2002b) and allows for generation of inferences connecting prior knowledge to narrative information (Hannon and Daneman, 2001; McNamara and Kendeou, 2011). Knowledge integration also allows comprehenders to update mental representations based on personal encounters and understandings (Long et al., 1989; Hamm and Hasher, 1992; Zwaan et al., 1995a; Prentice et al., 1997; Marmolejo-Ramos et al., 2009; Florit et al., 2011).

Narrative comprehension is vulnerable to deficits in prior knowledge (Graesser et al., 2003), particularly for children who have fewer world experiences (Graesser et al., 2002b). For example, consider the knowledgebase required to interpret a popular wordless picture book used to assess narrative comprehension processes in young children, *Frog, Where are You?* (Mayer, 1969). This 25-page picture book begins with an illustration of a boy and a dog in a bedroom. Also in the bedroom, a frog is in a jar. The child comprehender must first have some prior knowledge of, or experience with, animals kept in jars as pets. This knowledge must then be incorporated with the illustration to inform that the frog is the boy's pet. If this specific narrative event is new to the child, it will be more cognitively demanding to generalize and integrate prior knowledge (Graesser and Wiemer-Hastings, 1999) and potentially lead to difficulties in establishing a coherent understanding (Graesser et al., 2002b; Best et al., 2008). When successful, child comprehenders relate their own experiences to narrative content and appropriately infer meaning (Cain et al., 2001). For example, the child may know people hug animals that are pets and, therefore, infer from a picture of the boy hugging a frog that the frog is a pet. A child's knowledge-based inference for this event can potentially deepen comprehension, if successful, or limit them to a surface understanding if inference-making abilities are limited (Graesser and Kreuz, 1993; Graesser et al., 1997, 2002b; Graesser and Wiemer-Hastings, 1999; McNamara and Kendeou, 2011).

Despite the significance of knowledge integration for comprehension (Graesser et al., 1994, 1997, 2002b), this process has been investigated the least, particularly in children from low-income households (see Table 1). Our review of the literature found no studies intentionally assessing knowledge integration in children from low-income households, regardless of age. As such, there is little evidence to suggest these children integrate knowledge differently than children from middle- and high-income families; however, there may be qualitative differences due to varying background knowledge (McLoyd, 2013). For example, children from low-income households may be less familiar with concepts in a story about a preparatory school and exhibit less understanding of the character's goals than children from middle- or high-income families. On the other hand, they may have greater knowledge related to stories where characters independently overcome obstacles using their problem solving skills. Domain-specific knowledge studies (see Alexander et al., 1994 for a review), however, should not to be confused with investigating how generalized prior learning and experiences lead to inferences (Hannon and Daneman, 2001). The dearth of experimental manipulations of knowledge integration presents a significant gap in our knowledge of narrative comprehension. If converging research aims to provide a holistic view of children's comprehension, gaps surrounding this and other processes must be investigated across all populations.

### Goal structure understanding

Since narratives and everyday experiences follow goal directed patterns of actions and events (Trabasso, 1994; Berger, 1997), understanding links between characters' motives and narrative events is essential for forming coherent mental representations of narratives (Trabasso et al., 1989; Graesser et al., 1994, 1997; Wenner, 2004; Trabasso and Wiley, 2005; Lynch and van den Broek, 2007). Fundamental elements of narrative goal structure are goals, attempts, and outcomes (Trabasso and Nickels, 1992; Trabasso et al., 1992; Suh and Trabasso, 1993; Trabasso and Rodkin, 1994; Trabasso and Wiley, 2005; Lynch and van den Broek, 2007). Goals are defined as a character's desires that motivate subsequent actions (e.g., the boy wanted to eat). Attempts are actions taken to achieve the character's goal (e.g., the boy made a sandwich). Results of attempts are labeled outcomes, which can be successful, unsuccessful, reinstated, or abandoned. The degree that goal structure elements are logically matched facilitates comprehension in both adults and children (Albrecht and Myers, 1995; Low and Durrkin, 1998; Milch-Reich et al., 1999; Poynor and Morris, 2003; Egidi and Gerrig, 2006; Pyykkönen and Järvikivi, 2012; Orrantia et al., 2014).

Goal structure understanding also typically requires comprehenders to hierarchically relate goal structure elements (Trabasso and Nickels, 1992; Graesser et al., 1994; Trabasso and Wiley, 2005; Lynch and van den Broek, 2007). An initiating event causes an unwanted change in state for the protagonist (Trabasso et al., 1989) and a superordinate or primary goal forms to drive the remainder of the narrative (Trabasso and Nickels, 1992). Other goals supporting superordinate goal attainment are labeled “subordinate” and represented at lower levels of the hierarchy (Suh and Trabasso, 1993; Singer et al., 1994; Trabasso and Wiley, 2005). Subordinate goals are established when preliminary steps are required before an attempt can be made at the superordinate goal or when an attempt at a superordinate goal fails (Suh and Trabasso, 1993; Trabasso and Wiley, 2005; van den Broek et al., 2005). In the wordless picture book *Frog, Where are You?* (Mayer, 1969), children must understand that the boy's main, or superordinate goal, is to find the frog. In order to do so, the boy must form a subordinate goal of looking in specific locations (e.g., his boot, outside, in the woods; Trabasso and Rodkin, 1994). When an attempt successfully accomplishes a subordinate goal, another attempt can be made at the superordinate goal.

There is considerable agreement that understanding goal structures is important for comprehending narratives (Poynor and Morris, 2003; Lynch and van den Broek, 2007) through the generation of more inferences (Omanson et al., 1978; Lutz and Radvansky, 1997), aiding in retention when narratives are relatively long (Wenner, 2004), and allowing comprehenders to detect problems, anticipate solutions, and predict outcomes (Trabasso and Nickels, 1992). Goal structure understanding also increases understanding of main ideas (van den Broek et al., 2003). Few studies have investigated the impact of variations in goal structures. Research regarding the role of characters' competing goals, abandoned goals (Lutz and Radvansky, 1997; Magliano and Radvansky, 2001; McFarlan and Brown, unpublished manuscript), subordinate goals of secondary characters (Magliano et al., 2005), and multiple superordinate goals (Magliano and Radvansky, 2001; Linderholm et al., 2004) on comprehension is limited. Trabasso et al.'s (1992) work suggests that variations in objects that are targets of characters' motivations are important. Specifically, the frequent presence of a character's goal object in narrative scenes facilitates comprehension and may remind the comprehender of connections between character goals, goal objects, and attempts. Investigating goal structure variations will improve our current understanding of comprehension development and how goal structure understanding relates to causal inference generation.

### Causal inference generation

Causal inferences support knowledge integration and goal structure understanding by connecting time and place of actions, characters, character goals and motivations, internal states, and other narrative events (Trabasso et al., 1989; Suh and Trabasso, 1993; Trabasso and Suh, 1993; Graesser et al., 1994, 2001; Singer et al., 1994; Tompkins et al., 2013). Causal inferences fill narrative information gaps allowing comprehenders to integrate real-world knowledge with goal structure information (Trabasso et al., 1989; Graesser et al., 1994, 2001, 2002a; Tapiero et al., 2002), connect events across the goal structure (Trabasso et al., 1989; van den Broek, 1989; Trabasso and Nickels, 1992; Graesser et al., 1994; Zwaan and Radvansky, 1998; Tapiero et al., 2002; van den Broek et al., 2003; Brandão and Oakhill, 2005; Kendeou et al., 2008), and identify inconsistencies between narrative and mental representation (Long and Chong, 2001). In general, causal inferences promote recall (Trabasso et al., 1989; Bloom et al., 1990; McKoon and Ratcliff, 1992; Myers et al., 1994; Singer et al., 1994; Rizzella and O'Brien, 1996; van den Broek et al., 1996; Trabasso and Stein, 1997; Brownstein and Read, 2007) by organizing narrative events into causally related chains (Trabasso and van den Broek, 1985; Trabasso et al., 1989; Trabasso and Nickels, 1992; Myers et al., 1994; Singer et al., 1994; Wolman et al., 1997; Long and Chong, 2001; Tapiero et al., 2002).

Categorized by the logic and type of information connected, several taxonomies exist for describing causal inferences that assist the formation of coherent mental representations. One classification distinguishes local causal inferences that link proximal narrative content active in working memory (McKoon and Ratcliff, 1992; Graesser et al., 1994; Myers et al., 1994; Singer et al., 1994; Long and Chong, 2001; van den Broek et al., 2003) from global causal inferences that organize local narrative events into an established higher order (Myers et al., 1994; Singer et al., 1994; Long and Chong, 2001; Mason and Just, 2004; Brown et al., 2011). Another classification differentiates enabling, physical, motivational, and psychological inferences. Enabling inferences weakly relate narrative events by adding details and are considered least complex (Trabasso et al., 1989; Trabasso and Nickels, 1992). For example, “Max went up the stairs (antecedent). He heard a creaking noise (consequent).” Physical inferences establish physical causality between events and provide the strongest relations (Trabasso et al., 1989; Tapiero et al., 2002). For example, “The jar fell off the windowsill (antecedent). The jar shattered (consequent).” Motivational and psychological inferences are considered most complex (Trabasso et al., 1989). Motivational inferences connect characters' goals to narrative events (Trabasso and Nickels, 1992; Graesser et al., 1994). For example, “The boy wanted to catch the frog (antecedent). He chased after him (consequent).” Psychological inferences connect narrative events to characters' resulting internal states (i.e., emotions). For example, “The frog had gotten away (antecedent). The boy became very angry (consequent).” Although some inference types are more cognitively demanding than others, all ensure coherently organized mental representations form (Trabasso and Stein, 1997; Long and Chong, 2001; van den Broek et al., 2003).

While research has examined how inference generation relates to knowledge integration in the form of knowledge-based inferences (Nicholson and Imlach, 1981; Frank et al., 2003; Cain et al., 2004; Bowyer-Crane and Snowling, 2005; Shears et al., 2007), few studies examine this interaction in children (see Table 1). Some studies suggest knowledge of story structure, and from the narrative itself, inform knowledge-based inferences (Cain et al., 2001, 2004). Future research should intentionally examine knowledge that allows generation of these inferences in populations of young children (i.e., 1- to 4-year-olds). This would improve our estimation of children's understanding by distinguishing how cultural and developmental knowledge impacts inferences and comprehension.

### Summary

The constructionist approach to narrative comprehension has offered important information about processes underlying comprehension (Graesser et al., 1994, 1997; Kendeou et al., 2005, 2009) and has lead researchers to examine its application to describing development of narrative comprehension processes (e.g., Trabasso and Nickels, 1992; van den Broek et al., 2005; Lynch et al., 2008). In the next section, we review research on narrative comprehension development in young children and identify gaps in the extant literature.

## Narrative comprehension development

The last two decades have focused on applying the constructionist paradigm (Graesser et al., 1994) to children, non-readers, and reading achievement during school. Based on research with children from middle- and high-income households, we know that precursors to basic narrative comprehension processes emerge during infancy and reach mature levels around 9 years of age (Omanson et al., 1978; Bauer, 1992; Trabasso et al., 1992). As early as 8 months old, infants begin exhibiting immature causal inferences and goal structure understanding, such as sensitivity to causal structure and means-end (i.e., goal-attainment) problems in the real world (Sommerville and Woodward, 2005; Gerson and Woodward, 2012). By 20 months of age, children can generate enabling inferences and have limited recall of ordered events (Cohen et al., 1999; Wenner and Bauer, 1999). These studies of precursor processes support the constructionist paradigm's notion that, even in infancy, humans make sense of their world by searching for meaning (Franco, 1997).

Development of comprehension processes reaches a critical period between 3 and 5 years of age (Brown et al., 1986, 2011; Benson, 1997; Kendeou et al., 2005, 2008; van den Broek et al., 2005; Lynch et al., 2008; Tompkins et al., 2013). By the age of 3, children can occasionally generate inferences about causal relationships between isolated, physical objects when they encounter them in wordless picture narratives (Berman, 1988; Trabasso and Nickels, 1992; Trabasso et al., 1992; Brown et al., 2011). Children at this age rarely form coherent narrative representations (Berman, 1988) because they struggle to identify key goal structure elements (Trabasso and Nickels, 1992; Trabasso et al., 1992) and possess limited knowledge of the world (Kendeou et al., 2005). At 4 years old, children appear to be in developmental transition (Trabasso et al., 1992; Wenner, 2004). They become more sensitive to hierarchical goal structures and relations between events (Morrow, 1985; Trabasso and Nickels, 1992; Trabasso et al., 1992; van den Broek et al., 1996; Kendeou et al., 2005; Lynch and van den Broek, 2007; Brown et al., 2011). On the other hand, 4-year-olds rely more on enabling and physical inferences and less on complex inferences (van den Broek et al., 2005). Around age 5, children begin to use more mature processes (Trabasso and Nickels, 1992; Trabasso et al., 1992; Brown et al., 2011) and produce more goal-directed mental representations (Trabasso et al., 1992; Berman and Slobin, 1994; Kendeou et al., 2008; Brown et al., unpublished manuscript). These children have memorable experiences to integrate with narrative content, which increases the number and complexity of generated inferences (Trabasso and Nickels, 1992; Eaton et al., 1999; van den Broek et al., 2005; Brown et al., 2011).

From age 6 onward, comprehension processes continue to refine until maturity. Six-year-olds show increased sensitivity to causal relations (Lynch et al., 2008) and make more on-line inferences referring to superordinate and subordinate goals (Lynch and van den Broek, 2007). By age 7, children integrate world-knowledge and potentially over-rely on it for inference-making while ignoring narrative details (Cain et al., 2001; Brandão and Oakhill, 2005). Eight-year-olds appear to be more sensitive to subordinate goals and outcomes, but struggle with superordinate goals (van den Broek et al., 2003). Sensitivities to goal structures and inferences occurring at age 9 result in comprehension processing patterns similar to adult comprehenders (Trabasso et al., 1992; Orrantia et al., 2014).

### Future research directions

Our review of the literature has identified several important gaps in developmental research regarding narrative comprehension processes (see Table 1). One gap is research that intentionally assesses the process of knowledge integration in children. We assume children rely on this process as a component comprehension process (Pearson et al., 1979; Nicholson and Imlach, 1981; Fincher-Kiefer et al., 1988; Prentice et al., 1997; Cain et al., 2001), but there has been little effort devoted to describing its development. Additionally, few studies have examined goal structure understanding development in children younger than 4 years old (see Table 1). This gap is noteworthy given that children's narrative comprehension heavily depends on goal structure understanding (Low and Durrkin, 1998; Milch-Reich et al., 1999; Lynch and van den Broek, 2007; Pyykkönen and Järvikivi, 2012). Lastly, even less is known about early causal inference generation and goal structure understanding in children from low-income and minority populations. Curenton (2010) and Benson (1997) provide only limited information about causal inference generation and goal structure understanding in these populations of children. Research in this area has excluded populations struggling most in reading achievement outcomes (Federal Interagency Forum on Child and Family Statistics, 2013), and represents 74% of the lowest quartile on national reading assessments (NCES, 2011). Addressing these gaps will identify whether differences between populations exist and the responsible risk factors (e.g., socioeconomic status; McLoyd, 2013).

Underserved and underrepresented populations must be included in future examinations of how narrative variations inhibit or aid comprehension (e.g., Trabasso et al., 1992). van den Broek (1989) argued young children first make inferences between concrete events and are increasingly able to make inferences about abstract events as they age; however, no study has directly examined this or how children comprehend competing or abandoned goals in narratives. Investigating these variations, especially in children from low-income and minority households would extend our knowledge of comprehension, provide developmental standards for children's narratives, and set a more externally valid precedent for future research (Sue, 1999). The future and complexity of narrative research will also require a multi-method approach to assessing narrative comprehension processes.

## Narrative comprehension assessments

Narrative comprehension assessments typically belong to one of two categories: on-line and off-line (Berman, 1988; Trabasso and Nickels, 1992; Trabasso et al., 1992; Paris and Paris, 2003; Lynch and van den Broek, 2007). On-line assessments require responses be actively generated during narrative presentation whereas off-line assessments require reflective responses be generated after narrative presentation (Milch-Reich et al., 1999; Lynch and van den Broek, 2007). These categories of assessments provide different information about the multifaceted nature of comprehension.

### On-line assessments

On-line assessments measure what information is integrated into narrative mental representations in “real time” (Milch-Reich et al., 1999). As such, on-line assessments measure ongoing construction and maintenance of narrative mental representations (Renz et al., 2003). This type of assessment is often used with children because it requires less from their limited attentional resources (Milch-Reich et al., 1999; Lorch et al., 2010). Typical on-line assessments include think-aloud protocols, probe questions, and narrations (or storytelling).

Think-aloud protocols assess comprehension during narrative exposure by requiring on-going commentary indicating narrative understanding (Suh and Trabasso, 1993). Typically used for written text comprehension assessment in adults (Trabasso and Suh, 1993; Magliano et al., 1999; Kendeou et al., 2011), think-aloud protocols have informed how and when mental representations form and update (Graesser et al., 1997; Kurby and Zacks, 2012). When used with children as young as 6 years old, narrative events are presented as picture books and children describe the main character's thoughts (Lynch and van den Broek, 2007) or what is happening in the scene (Milch-Reich et al., 1999). Think-aloud protocols have the potential to assess how comprehension processes are developing online at different ages (Suh and Trabasso, 1993; Milch-Reich et al., 1999; Lynch and van den Broek, 2007). Nevertheless, this method must be adjusted to assess young children who have limited or developing expressive vocabularies (Lynch and van den Broek, 2007).

Probe questions are open-ended questions assessing comprehenders' current mental representation at a given point during narrative exposure (Lutz and Radvansky, 1997; Lorch et al., 2006). A probe question might ask a child, “Why is the boy mad at his parents?” Answering requires recalling narrative events and generating inferences. Responses are analyzed for accuracy and are indicators of comprehension processes (e.g., goal-related inferences; Lynch and van den Broek, 2007). Probe questions are challenging for younger comprehenders as they interrupt and divert children's already limited attentional resources and may actually disrupt comprehension (van den Broek et al., 2001).

Story narration methods typically require comprehenders create an oral story based on picture sequences (Berman and Slobin, 1994; Berman, 1995, 2004). Story narrations are perhaps most often employed using wordless picture books when assessing comprehension processing in preschool children (Shapiro and Hudson, 1991; Trabasso and Nickels, 1992; Paris and Paris, 2003; Brown et al., 2011). Picture books are often used because the stationary images offer fewer distractions for child comprehenders (Pike et al., 2010). Arfé and Boscolo (2006) asked a sample of hearing and non-hearing children to write, rather than orally produce, a story based on the children's picture book *Frog, Where are You?* (Mayer, 1969). Resulting narrations are analyzed for words used (Berman, 1988; Pelletier and Astington, 2004), number of goal related inferences generated (Lynch and van den Broek, 2007), accuracy of identified goal structure elements (Pemberton and Watkins, 1987; Brown et al., unpublished manuscript), and frequency and complexity of causal inferences generated (Arfé and Boscolo, 2006; Brown et al., 2011). Narrations allow individual processes to be assessed in terms of maturation. Ideal narrations include purposeful organization of narrative events (i.e., goal structure understanding), causal inferences of varying complexity, and integration of world and narrative knowledge (Trabasso and Nickels, 1992; Trabasso et al., 1992).

Other measures such as brain imaging, eye tracking, and reading times have also been used to assess on-line processes. Event related potentials (ERPs) and eye tracking have been employed in examinations of knowledge integration (Cook and Myers, 2004; Ferretti et al., 2013; Filik and Leuthold, 2013). Specific brain areas have been identified as important for causal inference generation (Mason and Just, 2004). Orrantia et al. (2014) showed 11-year-olds were more efficient than 9-year-olds at connecting character goals with actions based on faster reading times (see also Albrecht and Myers, 1995). Other studies use reading times to compare the availability of neutral, completed or achieved, and failed goal information in memory (Lutz and Radvansky, 1997; Richards and Singer, 2001). Obviously, reading times are inappropriate for young non-readers; however, eye-tracking methods measuring looking times during picture book narrations may provide insight into processing that emerges prior to formal reading (Evans and Saint-Aubin, 2005). Although ERPs have assessed language development in young children (e.g., Friederici, 2005; see Kuhl, 2010), brain imaging has not been extended to investigations of on-line narrative comprehension processing in preschool-age children.

### Off-line assessments

Off-line assessments measure the outcome of processing and what information has been included in comprehenders' final mental representations (Milch-Reich et al., 1999; Lynch and van den Broek, 2007). Although these assessments may not be the most appropriate for young children (Gibbons et al., 1986; Tompkins et al., 2013), they identify specific narrative content that has been comprehended. Commonly used off-line assessments include free recall, narrative retellings, and cued recall.

Free recall tasks assess what content is encoded as most important and accessible in a narrative (Kendeou et al., 2005). Open-ended recall prompts often ask comprehenders to “tell what you can remember from the story.” This method allows for large variations in responses and has been instrumental in identifying individual and developmental differences in recalled content (van den Broek et al., 1996; Lorch et al., 2010). Free recall has examined comprehension across different narratives media types (e.g., videos, written text, and aural stories; Kendeou et al., 2005, 2008), how much narrative information was remembered (Kendeou et al., 2008, 2009; Kim et al., 2008) and comprehenders' causal sensitivity (Tompkins et al., 2013). Because of demands placed on attention, memory and interest, simple free recall tasks are not as sensitive to young children's comprehension (Gibbons et al., 1986).

Narrative retellings, a form of free recall, are considered the most ideal off-line assessment for child populations, as they allow them to revisit their narrative experience (Morrow, 1985; Wenner, 2004). Retellings may take the form of a verbal story (Trabasso et al., 1992; Lorch et al., 2010) or physical enactment with or without props (Morrow, 1985; Wenner, 2004). Accuracy of children's retellings indicates their sensitivity to narrative goal structure and inferred event relations (Morrow, 1985). Murachver et al. (1996) found the use of props and characters while enacting the narrative increased children's comprehension of narrative events. They suggest actively involving children in the narrative may assist comprehension by highlighting goal and attempt relations (Murachver et al., 1996).

Compared to free recall, cued recall is useful for determining the most effective cues for retrieving information from mental representations (Paris et al., 1977). Lorch et al. (2006) used cued recall questions to assess children's comprehension of narrative events. They found children's accuracy was greatest for narrative events in causal chains. Unlike on-line probe questions, cued recall questions typically yield very literal, content-based answers when used with children (Lynch and van den Broek, 2007). However, Omanson et al. (1978) found increased inferential comprehension for 5- and 8-year-olds during cued recall when compared to free recall. Cued recall may encourage inferences through vague questions such as, “What made the boy leave his house?” or assess specific narrative understanding through more literal questioning, such as “Who was in the tree?” (Lorch et al., 1999a). These questions draw attention to central narrative details, encourage inference generation, and assist in young comprehenders' maintenance of information (Lorch et al., 1999a,b, 2000, 2004, 2006, 2010; Curenton, 2010). As considerable dialog between early childhood educators and their students already involves inferential questioning (Zucker et al., 2010), researchers have increasingly employed cued recall to assess narrative comprehension in school-age children.

### Comparing and combining assessments

Accurately assessing comprehension demands a multi-method approach be taken, particularly when assessing narrative comprehension processes in young children. Our previous descriptions of on- and off-line assessments highlight knowledge added from each assessment and its developmental appropriateness. However, both on- and off-line assessments contain methodological aspects that limit their use in isolation.

Investigating young children's comprehension processes typically employs on-line comprehension measures (e.g., picture book narrations; Berman, 1988; Trabasso and Nickels, 1992; Trabasso et al., 1992; Paris and Paris, 2003; Lynch and van den Broek, 2007; Brown et al., 2011). Although efficient and frequently used, narrating picture books may underestimate children's competencies if used in isolation (Trabasso et al., 1992). Three- to 5-year-olds, for example, may not fully articulate their understanding due to immature expressive language (Berman, 1988, 2004; Shapiro and Hudson, 1991; Berman and Slobin, 1994; Wigglesworth, 1997; Pearce, 2003; Kulkofsky et al., 2008; Curenton, 2010) and would benefit from additional comprehension tasks. Similarly, young children require additional training procedures to complete think-aloud protocols (Lynch and van den Broek, 2007). Isolated assessments may be unable to differentiate between cognitive resources used for completing the task (e.g., vocabulary) and specific comprehension processes (e.g., causal inference generation). Graesser et al. (1997) further argue that some on-line assessments, such as reading times, may provide ambiguous evidence about processes involved.

Similarly, off-line measures should not be used in isolation with children who may not possess the cognitive maturity required to construct a complete representation. Specifically, young children's limited attentional and memory resources may negatively impact performance on off-line assessments (Lorch et al., 1999a,b, 2000, 2004, 2006, 2010; Milch-Reich et al., 1999). While some off-line assessments use support props or illustrations (Morrow, 1985; Murachver et al., 1996; Wenner, 2004), narratives unable to provide such accommodations risk children misunderstanding story content. Children's comprehension errors made during narrative exposure are not easily corrected after their mental representation is constructed (van den Broek et al., 2001). As comprehension processing development is central to children's reading outcomes (Benson, 1997; van den Broek et al., 2005; Lynch and van den Broek, 2007; Kendeou et al., 2008; Brown et al., 2011), measures with developmental limitations cannot be used in isolation. We propose on- and off-line measures used in conjunction may provide insight as to how children engage specific processes when forming coherent mental representations.

A limited set of studies underscores the significance of a multi-method approach to understanding narrative comprehension development. van den Broek et al. (2005) suggested cued recall, in addition to story narrations, may provide information regarding young children's narrative mental representations. An early study employing this multi-method approach measured comprehension using cued and free recall but did not find parallel comprehension increases for 5- and 8-year-olds (Omanson et al., 1978). However, more recent studies found 4- to 8-year-olds' inferences generated on-line during think-aloud protocols and story narration positively related to the amount of narrative information included in both free and cued recall (Lynch and van den Broek, 2007; Tompkins et al., 2013). This suggests specific method combinations may provide accurate depictions of children's mental representations. For example, Trabasso et al. (1992) used both story narrations and cued recall questions to assess children and found, when prompted by cued recall, increases in story comprehension.

A multi-method approach to narrative comprehension processes research also has the potential to address many of the gaps in the extant literature. One gap is the examination of comprehension processes across narrative media presentations (Kendeou et al., 2005, 2008). The constructionist paradigm (Graesser et al., 1994) asserts processing should be similar regardless of presentation, which limited research has confirmed. Another gap encompasses the development of knowledge integration. Research suggests combining on- and off-line measures is most informative for investigating knowledge integration with adults. Narvaez et al. (1999) found changes in comprehenders' purpose (i.e., entertainment vs. studying) led to differences in on-line processing measured by think-aloud protocols, but not in off-line processing measured by free recall. Specifically, comprehenders' were more likely to engage in knowledge integration when reading to study. Thus, comprehenders' intentions may impact knowledge integration during on-line comprehension, but not the final mental representation. This finding has implications for reading instruction. Through intentional selection of multi-method assessments that can address variations in individual knowledge, population-based differences, and narratives across media, the body of comprehension research will more accurately describe how processes develop in all children.

## External validity and narrative comprehension development research

Review of the current literature suggests an emphasis on internal validity when assessing narrative comprehension development that has resulted in a de-emphasis on external validity (Anderson et al., 1999; Sue, 1999). Internal validity addresses whether the construct being measured (e.g., tiredness) causes a specific effect (e.g., crankiness); whereas external validity addresses whether a causal relationship can be generalized across other measures, populations, time, and settings (e.g., Does tiredness make all children cranky? Bracht and Glass, 1968; Calder et al., 1982). These validities have an inverse relationship, such that increasing experimental control (internal validity) decreases generalizability (external validity). Arguments against externally valid studies include the suggestion that such studies are nearly impossible to conduct (Calder et al., 1982; Mook, 1983) and that they decrease internal validity, which hinders progress of scientific research (Calder et al., 1982). Alternatively, it has been suggested that subtle, systemic biases have crafted contemporary psychology to value empiricism and internal validity, resulting in a lack of high quality ethnic minority research (Sue, 1999). Despite resistance to, and perceived difficulty of, conducting experiments that account for background factors affecting generalizability (Calder et al., 1982), externally valid research has been influential in identifying significant truths about how humans operate (Anderson et al., 1999; Quintana et al., 2006). For example, Paris and Paris (2003) assessed on- and off-line narrative comprehension in 158 racially and socioeconomically diverse 5- to 8-year-olds from the same city. Despite having a representative sample, they reported finding only developmental and ability-related differences in children's comprehension rather than differences related to racial and socioeconomic factors. These results raise the empirical question of whether demographics systematically relate to narrative comprehension abilities (Sue, 1999; McLoyd, 2013). Addressing this requires first identifying if significant differences stem from race or socioeconomic status, and then under what circumstances those differences manifest. However, until greater research intentionally assesses diverse populations, we can only speculate differences in background knowledge may exist. Hence, we have highlighted external validity concerns in the extant literature and identified what may be gained by addressing these concerns. Specifically, we argue for more studies that account for individual knowledgebases, differences in populations, and narrative media types employed when assessing young children.

### Individual knowledgebase

In order to broaden the scope of narrative comprehension research to include all children, researchers must proactively consider the impact of individual differences in knowledgebase on comprehension. It is understood that knowledge impacts mental representation formation (Myers et al., 1994; Singer et al., 1994; Zwaan et al., 1995a,b; Long and Chong, 2001; Brandão and Oakhill, 2005; Gerrig, 2011; Kurby and Zacks, 2012) and comprehension (Gowie, 1973; Graesser et al., 1994, 1997; Best et al., 2008); however, knowledgebase content is constrained by many external factors including age, gender, environment, geography, culture, race and ethnicity, and socioeconomic status. The problem arises when highly controlled experiments find deficits in children's comprehension processes that can be attributed to individual variations in knowledge. For example, a child from a metropolitan area may not have the necessary knowledge to integrate and comprehend why the boy would want to capture and bring home a frog in the picture book *Frog, Where are You?* (Mayer, 1969). Labeling such variations as merely individual differences (Hannon and Daneman, 2001) is problematic because it implies that a standard body of knowledge transcends all ages, cultures, and differences; and that any knowledge deviations are indications of cognitive deficits.

We must consider more directly then the impact of knowledgebase differences on narrative comprehension processes and their assessment (Graesser et al., 1997). When assessing different cohorts of children, Berman (2004) noted that the concept of a “birthday party” differed for American and Israeli preschoolers. For American children, a birthday party was typically an open-ended script. For Israeli preschoolers, however, it was associated with a highly conventionalized and stereotyped concept. This difference in knowledge may impact goal structure understanding, causal inference generation, and overall comprehension for narratives that include birthday party information.

Differences in experiences may affect what knowledge comprehenders integrate during comprehension (Berman and Slobin, 1994; Gorman et al., 2011). A robust literature describes the importance and frequency of storytelling interactions in African American and low-income families (Gardner-Neblett et al., 2012). There is some suggestion that African American children from low-income households may actually have unique strengths in narrative processing (Gardner-Neblett et al., 2012) because storytelling practices provide children with early exposure to narrative structure and rules (Sperry and Sperry, 1996). Indeed, Curenton (2010) found that, among samples from low-income families, African American children understood characters' goals more often than European American children.

Future comprehension research must select narrative stimuli, assessments, and study designs that account for knowledgebase. For example, Hannon and Daneman (2001) provided nonsense concepts that related to real-world images (e.g., a *MIRT* resembles an ostrich, but with a larger and longer neck), which measured whether participants had access to specific prior knowledge that impacted their inference making. Performance on this task accounted for much of the variance in reading comprehension, suggesting prior knowledge had a significant impact on comprehension. Assessing knowledge used during specific comprehension tasks can reduce biases in future research that may be due to environmental, economic, or cultural variations (see Sue, 1999; McLoyd, 2013). If researchers intend to close existing gaps in the literature, changes in experimental procedures must be made to account for variations in knowledgebase across diverse populations (Gorman et al., 2011).

### Diverse populations

By examining narrative comprehension development in diverse populations, researchers will better understand the development of fundamental comprehension processes. Much of the reviewed literature has indicated a trend for assessing convenient and relatively homogenous samples (Sue, 1999). In reality, many studies fail to specify the sample and population (e.g., Cain et al., 2004; Kendeou et al., 2008, 2009). This is detrimental in that much can be gained from examining how ordinary human variations impact comprehension processing. We have identified how diverse samples informed previous narrative comprehension research through differences in ability, culture, and environment; yet, a dearth of research directly tests the role of these factors.

Examining comprehension processes in populations with diverse cognitive abilities has enhanced our general understanding of resources necessary for comprehension. For example, numerous examinations of children with attention deficit hyperactivity disorder (ADHD) have advanced our appreciation for attentional resources needed for narrative comprehension and young comprehenders' limits (Tannock et al., 1993; Lorch et al., 1999a,b, 2000, 2004, 2006, 2010; Renz et al., 2003). One study examining children with mild mental retardation and learning disabilities found narrative recall was related to information on causal chains (Wolman et al., 1997). This confirmed that children's and adults' cognitive load is reduced when comprehending narratives that are highly causally-related (Trabasso and Sperry, 1985; Trabasso and van den Broek, 1985; van den Broek et al., 1996; Lorch et al., 2006). Similar to typically developing children (Kendeou et al., 2008), this sensitivity to causal structure has been found to develop relatively independently of basic language skills in young children with Down syndrome (Kim et al., 2008). Investigations of non-hearing (Arfé and Boscolo, 2006; Chamberlain and Mayberry, 2008) and non-seeing (Carreiras and Alvarez, 1999) populations have demonstrated both similarities and differences in narrative comprehension processing. These studies speak to factors supporting the development of comprehension processes and highlight the necessity of examining populations typically underrepresented in research.

A greater concern regarding the current body of literature is the underrepresentation of racial and ethnic minority samples. Findings from predominately European American, middle-class populations may yield results with minimal variability and limited generalizability to other populations (Sue, 1999; Frierson et al., 2008). It is suggested that there are numerous concerns to be addressed when recruiting racial and ethnic minorities in research (Frierson et al., 2008). A limited number of studies have focused on traditionally underrepresented and underserved populations when examining narrative comprehension processes. (Melzi, 2000; Fiorentino and Howe, 2004; Curenton, 2010; Gorman et al., 2011; Brown et al., unpublished manuscript). Results from Gorman et al., (2011) identified cultural differences in storytelling style and how these differences impact comprehension. Latino children emphasized character names during storytelling, African American children included story embellishments, and European American children emphasized character relationships. While this procedure provided a culturally non-biased context for analyzing story production, a less culturally sensitive researcher might have concluded that some children struggled to identify and emphasize key story elements based on stylistic differences. It is imperative then that future studies consider the role of cultural values for future and past narrative research (Quintana et al., 2006).

Some research intentionally increases external validity by purposefully including children from low-income and racial and ethnic minority populations in samples (e.g., Curenton, 2010; Brown et al., unpublished manuscript). As a result of their focus, these researchers are regularly challenged and criticized about the validity and necessity of their work (Sue, 1999). Common criticisms highlight that these studies may lack control groups of children from majority or middle-income populations. Further, there is reluctance to include such studies as part of converging evidence about typical developmental trends. Although these arguments suggest a desire to maintain basic experimental control and internal validity, they have made research of underrepresented groups difficult to conduct and fund (Sue, 1999). This suggests the desire to conduct externally valid research exists, but is met with resistance by the scientific community.

Since early comprehension processes are strong predictors of later comprehension and reading skills (van den Broek et al., 2001, 2003, 2005; Brown et al., 2011), it is essential to include children placed at-risk using externally valid assessments (Benson, 1997; Brown et al., unpublished manuscript). Despite this, the current review identified the dearth of such research (see Table 1). As at-risk communities typically experience threats to development during critical learning periods, less access to health-care and resources, and diverse values (Morrow, 1985; Bradley and Corwyn, 2002; Evans, 2004; Curenton, 2010), there may be differences in knowledgebases used to form coherent narrative mental representations (Sharp et al., 1995). It has also been suggested that children from low-income communities have difficulty generating narratives and require additional attention in schools (Fiorentino and Howe, 2004). As a considerable portion of comprehension research has examined children's narrative comprehension through story narration, these findings suggest a new approach may be necessary. For example, future comprehension assessments using familiar or dynamic narrative stimuli (e.g., televised narratives, multimedia books) may provide support to children who struggle with narrative production (Sharp et al., 1995; Wright et al., 2001; Verhallen et al., 2006). We assert then that future research must accommodate and include people from underrepresented groups, particularly children. Using a multi-method approach to assess comprehension will ensure these populations are accurately evaluated. More importantly, though, they will receive the same attention that has identified comprehension difficulties in typically measured populations.

### Media type

While knowledge, experiential, and cultural differences may impact children's narrative comprehension processing, the constructionist paradigm argues that these underlying processes are generalizable across narrative media type (Graesser et al., 1994; Kendeou et al., 2005, 2008, 2009). This idea is supported by fMRI investigations by Anderson et al. (2006) that found that comprehending silent filmic montages activated brain regions similar to those activated by comprehending language and narratives. This suggests that comprehension of narrative-structured events recruit similar cortical networks regardless of presentation. These results were confirmed by Kendeou et al. (2008) who found 4- and 6-year-olds' inference generation were interrelated across aural, written, and televised stories. This interdependency continued over time as children turned 6 and 8 years old. Thus, as society becomes increasingly technological, it is necessary that research continue to examine this generalizability of comprehension processing across narrative media during children's development (Anderson and Hanson, 2009; Christakis and Zimmerman, 2009). The Kaiser Family Foundation (Rideout and Hamel, 2006) reported that 81–87% of 2- to 6-year-olds read or are read to everyday, but more than 70% also watch television daily. Further, more than 40% of young children spend 2 hours or more watching television in a typical day and 29–43% have television in their bedroom. Parallel changes in narrative stimuli formats must be considered for the future of comprehension research in order to ensure its external validity.

Still, narrative research often selects wordless picture books as stimuli for young children because they limit distractors and require basic comprehension processes (Pike et al., 2010). This may also be partially due to public resistance toward television and the argument that it negatively impacts cognitive and social development (Vandewater et al., 2007; Schmidt et al., 2008; Kirkorian et al., 2009). A growing body of research, however, refutes this assertion and suggests that regulating the amount of media exposure and content may actually benefit and educate children (Schmidt and Vandewater, 2008; Kirkorian and Anderson, 2009; Anderson and Hanson, 2009; Kirkorian et al., 2009). For example, educational programs such as *Sesame Street* and *Blues Clues* use goal-oriented narratives to discuss topics later covered in schools and to teach problem solving (Kirkorian et al., 2008). The promise of such programming suggests that narrative media may be beneficial in assessing and improving children's comprehension processing.

It seems apparent then that both developmental appropriateness and potential benefits of media narratives must be considered for the future of comprehension research. For example, dynamic conventions associated with televised narratives for children (e.g., scene changes, transitions, off-screen audio) may be too complex for children younger than 24 months (Anderson and Hanson, 2009, 2010; Kirkorian et al., 2009, 2012; Pempek et al., 2010). Alternatively, it has been suggested that elaborate visual information enhances comprehension processing in young children (Shapiro and Hudson, 1991; Pearce, 2003; Orrantia et al., 2014). By 3 years old, it appears that children can discriminate between symbolic representations of the world and the real world efficiently enough to engage in instructional problem solving (e.g., 3-year-olds can watch a video of a toy hidden in a room, and later locate the hidden toy when brought into a room identical to the depiction; Schmitt and Anderson, 2002). As an extension, it is possible that dynamic visual information accompanied by auditory information available in television has greater benefits to comprehension processes as children mature. Indeed, storybooks presented in a multimedia format were found to improve causal inference generation, narrative retellings, and overall coherence in 5-year-olds placed at-risk compared to storybooks with static pictures (Verhallen et al., 2006). These studies suggest that, while comprehension processes generalize across media formats, certain populations may benefit from different or enhanced narrative presentations.

When contemplating the future of comprehension research, it is important to change opinions regarding narrative media in order to identify and maximize benefits for children (Anderson and Hanson, 2009; Christakis and Zimmerman, 2009). Using non-traditional narrative media presentations, such as television or interactive “e-books,” may reduce cognitive load, improve recall of narrative events, and enhance story comprehension for children (Gibbons et al., 1986; Sharp et al., 1995; Linebarger and Piotrowski, 2009; Korat, 2010). As an increasingly technological society, researchers must consider the ecological validity of laboratory stimuli (Pearce, 2003). This is particularly important for children from underrepresented communities who often have more access to televisions than print media (Sharp et al., 1995; Neuman and Celano, 2001; Evans, 2004; Rideout and Hamel, 2006). It is imperative that narrative stimuli be developmentally and ecologically appropriate for all children's comprehension processes.

## Conclusion

This review argued for intentional changes to increase the external validity of narrative comprehension development research. Pervasive internal validity emphasis within the scientific community has deemphasized external validity and led to unbalanced research practices (Sue, 1999). This endeavor requires future studies employ externally valid rationales in order to fill important gaps in the current literature. An intentional shift toward balancing converging evidence with internally and externally valid studies will ensure accurate assessment of future children's comprehension. As a research area with significant academic implications, future work must include traditionally understudied and underserved populations (Sharp et al., 1995; Sue, 1999; Neuman and Celano, 2001; Evans, 2004; Rideout and Hamel, 2006) who continue to struggle in reading achievement (NCES, 2011; Federal Interagency Forum on Child and Family Statistics, 2013). This will require the intentional inclusion of diverse populations and increase in cultural validity of laboratory studies (Sue, 1999; Quintana et al., 2006; McLoyd, 2013).

The theoretical framework of this review, the constructionist paradigm (Graesser et al., 1994), lends itself to applied future studies that would improve the generalizability of converging comprehension research. Therefore, intentional inclusion of underserved and underrepresented children in future studies will provide a more accurate, holistic view of and how environmental factors contribute to comprehension development. Accompanying this inclusion, researchers must be mindful of differences in knowledgebases when creating age and culturally appropriate narratives for assessment. This, in turn, will offer clearer insight into improving assessments for underserved populations (e.g., using narrative stimuli that are sample-appropriate and ecologically valid). Through the purposeful investigation of these populations, improved comprehension measures will be developed to benefit all children.

We would be misguided to ignore societal changes that impact child development, and must, therefore, adapt methodology to assess narrative comprehension in the current era (Anderson and Hanson, 2009; Christakis and Zimmerman, 2009). Indeed, policies and practices within research laboratories must also reflect this (McLoyd, 2013). The constructionist paradigm of narrative comprehension has the potential to explain comprehension through previously excluded narrative stimuli (Graesser et al., 1994; Kendeou et al., 2005, 2008, 2009). The outcomes of such changes remain an empirical question similar to ideas concerning generalizability across populations (Sue, 1999; Brown et al., unpublished manuscript). Broadening the scope of narrative comprehension research will only have positive implications for academic and societal outcomes. When research balances internal and external validity we will be able to truly assess when, and how, all children comprehend.

### Conflict of interest statement

The authors declare that the research was conducted in the absence of any commercial or financial relationships that could be construed as a potential conflict of interest.

## Appendix A

### Description of table 1

Table 1 describes 71 sources observing comprehension processes in children. Some studies did not explicitly identify the comprehension processes as described in this paper. In such cases, studies assessing constructs such as “causal relatedness,” “inference-making,” and “relations between events” were understood to be measuring causal inference generation. Constructs such as “event relatedness,” “goal actions,” and “intentionality” were understood to be measuring goal structure understanding. “World knowledge,” “general knowledge,” and “generalization of knowledge” were understood to be measuring knowledge integration. Table 1 also distinguishes these processes according to sample-income. When not made explicit, maternal and paternal education means at, or above, 16 years were considered proxies for middle-income status. Studies without indicators of socioeconomic status were assumed to have examined middle- to high-income samples and are noted in Appendix B by an asterisk (^*^).

## Appendix B

Sources Describing Narrative Comprehension Processes Across Low- and Middle- to High-Income Households.

**Studies of Child Samples Representing Lower-Income Households:**

**Assessing Goal Structure Understanding**

1. Benson, 1997
2. Brown et al., unpublished manuscript
3. Eaton et al., 1999
4. Gorman et al., 2011
5. Linebarger and Piotrowski, 2009
6. Morrow, 1985
7. Orrantia et al., 2014
8. Paris and Paris, 2003
9. Pelletier and Astington, 2004
10. Tompkins et al., 2013
11. Verhallen et al., 2006

**Assessing Causal Inference Generation**

1. Benson, 1997
2. Florit et al., 2011
3. Milch-Reich et al., 1999
4. Pelletier and Astington, 2004
5. Sperry and Sperry, 1996
6. Tompkins et al., 2013
7. Zucker et al., 2010

**Studies of Child Samples Representing Middle-Income to High-Income Households^*^:**

**Assessing Knowledge Integration**

1. Best et al., 2008
2. Bowyer-Crane and Snowling, 2005^*^
3. Brandão and Oakhill, 2005^*^
4. Cain et al., 2001^*^
5. Long et al., 1989^*^
6. McNamara et al., 1996^*^
7. Murachver et al., 1996
8. Nicholson and Imlach, 1981^*^
9. Pearson et al., 1979

**Assessing Goal Structure Understanding**

1. Aldrich et al., 2011^*^
2. Bauer et al., 1999
3. Bauer and Shore, 1987^*^
4. Berman, 1988^*^
5. Berman, 1995^*^
6. Berman, 2004^*^
7. Berman and Slobin, 1994^*^
8. Brown et al., 1986^*^
9. Cain et al., 2004^*^
10. Eaton et al., 1999
11. Henderson and Woodward, 2011^*^
12. Kirkorian et al., 2012^*^
13. Lorch et al., 2006^*^
14. Lorch et al., 2010^*^
15. Lorch et al., 1999b^*^
16. Low and Durrkin, 1998
17. Lynch and van den Broek, 2007
18. Lynch et al., 2008
19. McCabe et al., 2008
20. McNamara et al., 1996^*^
21. Morrow, 1985
22. Murachver et al., 1996
23. Omanson et al., 1978^*^
24. Pearce, 2003^*^
25. Pelletier and Astington, 2004
26. Pyykkönen and Järvikivi, 2012^*^
27. Renz et al., 2003^*^
28. Sommerville and Woodward, 2005^*^
29. Tannock et al., 1993^*^
30. Tompkins et al., 2013
31. Trabasso and Nickels, 1992
32. Trabasso et al., 1992
33. van den Broek et al., 2003^*^
34. van den Broek et al., 1996^*^
35. Wenner, 2004
36. Wenner and Bauer, 1999
37. Wigglesworth, 1997

**Assessing Causal Inference Generation**

1. Bauer, 1992^*^
2. Bauer et al., 1998
3. Bauer et al., 1999
4. Bauer and Shore, 1987^*^
5. Brandão and Oakhill, 2005^*^
6. Brown et al., 2011
7. Cain et al., 2001^*^
8. Cain et al., 2004^*^
9. Cohen et al., 1999^*^
10. Florit et al., 2011
11. Gibbons et al., 1986^*^
12. Henderson and Woodward, 2011^*^
13. Kendeou et al., 2008^*^
14. Kendeou et al., 2009^*^
15. Kim et al., 2008^*^
16. Kulkofsky et al., 2008
17. Lorch et al., 1999a^*^
18. Lorch et al., 2004^*^
19. Lorch et al., 2010^*^
20. Lorch et al., 2000^*^
21. Lorch et al., 1999b^*^
22. Low and Durrkin, 1998
23. McNamara et al., 1996^*^
24. Milch-Reich et al., 1999
25. Omanson et al., 1978^*^
26. Pelletier and Astington, 2004
27. Pike et al., 2010^*^
28. Schulz and Gopnik, 2004
29. Tompkins et al., 2013
30. van den Broek et al., 2003^*^
31. van den Broek et al., 1996^*^
32. Wenner, 2004
33. Wenner and Bauer, 1999
34. Wolman et al., 1997

Studies without indicators of socioeconomic status were assumed to have examined middle- to high-income samples and are noted here by an asterisk (^*^).
